# Epithelial Ovarian Cancer: The Role of Cell Cycle Genes in the Different Histotypes

**DOI:** 10.2174/1874189400802010007

**Published:** 2008-02-06

**Authors:** Giuseppina D’Andrilli, Antonio Giordano, Alessandro Bovicelli

**Affiliations:** 1 Department of Obstetrics and Gynecology, Sant’Orsola Hospital, University of Bologna, 40138 Bologna, Italy; 2 Sbarro Institute for Cancer Research and Molecular Medicine, Dept. of Biology, College of Science and Technology, Temple University, Philadelphia, PA 19122, USA; 3 Department of Human Pathology and Oncology, University of Siena, Siena, Italy

**Keywords:** Cell cycle deregulation, ovarian malignancy, cancer therapy, ovarian carcinoma histotypes

## Abstract

Cancer is frequently considered to be a disease of the cell cycle; alterations in different families of cell cycle regulators cooperate in tumor development. Molecular analysis of human tumors has shown that cell cycle regulators are frequently mutated in human neoplasms, which underscores how important the maintenance of cell cycle commitment is in the prevention of human cancer. The regulatory pathways controlling cell cycle phases include several oncogenes and tumor suppressor genes which display a range of abnormalities with potential usefulness as markers of evolution or treatment response in epithelial ovarian cancer. This review summarizes the current knowledge about these aberrations in malignant tumors of the ovary. We sought to focus our attention on the genes involved in the development of tumors arising from the ovarian epithelium, which are the most common types of ovarian malignancies.

## INTRODUCTION

Ovarian cancer remains a highly lethal disease. In developed countries, ovarian cancer accounts for more deaths than all other gynecological malignancies combined; an estimated 22,430 new cases and 15,280 deaths occurred in 2007 (Cancer Facts & Figures 2007). As the result of advances in surgical management and chemotherapeutic options over the last three decades, the median survival times for ovarian cancer patients has improved. However, overall survival has not been significantly changed. In addition, most patients present with advanced disease for which highly effective curative therapy is currently unavailable. The epithelial tumors of the ovary, which account for 90% of malignant ovarian tumors, are further divided into histological subgroups with different malignant potential endometrioid, mucinous, serous, clear cell and undifferentiated carcinomas. In follow-up studies, mucinous and endometrioid carcinomas have a less aggressive behavior and a better overall survival than serous tumors. A characteristic of serous, mucinous and endometrioid ovarian carcinomas is the low malignant potential (LMP), or in the case of borderline tumors, a low risk of invasion.

The molecular pathology of ovarian carcinomas is heterogeneous and involves various putative precursor lesions and multiple pathways of development. The most common subtype, high-grade serous carcinoma, is characterized by p53 mutations, and BRCA1 and/or BRCA2 dysfunction. It most likely arises from epithelium within inclusion cysts or from the surface of the ovary. Dysregulation of cell cycle control, in particular G_1_-S-phase transition, is implicated in the pathogenesis of most human cancers, including epithelial ovarian cancer (EOC). However, the prognostic significance of aberrant cell cycle gene expression in EOC remains mostly unclear. During the G_1_-S transition, the cy-clinE/CDK2 and cyclinD/CDK4 complexes promote progression and are each inhibited by the associated CDK inhibitor p27^KIP1^. If DNA damage occurs, p53 accumulates in the cells and induces the p21-mediated inhibition of cy-clinD/CDK. The transition to S phase is triggered by the activation of the cyclinD/CDK complex, which phosphory-lates the retinoblastoma protein pRb, a known cell proliferation regulator.

The members of the INK4 family, comprising p16 and p15, exert their inhibitory activity by binding to the CDK4 and CDK6 kinases and preventing their association with D-type cyclins.

In contrast to G_1_ regulators, less is known about the genes which regulate the S, G_2_ and M phases of the cell cycle, such as cyclin A- and cyclin B-kinase complexes and their inhibitors. The significance of cell-cycle regulatory genes in carcinogenesis is underlined by the fact that most of them have been identified as proto-oncogenes or tumor suppressor genes.

Expression of cell cycle markers has recently been linked to tumor behavior and response to treatment. Hopefully, a better understanding of the molecular mechanisms underlying the tumorigenic process of ovarian carcinoma will lead to earlier diagnosis, novel therapies and ultimately better outcomes.

## SEROUS OVARIAN CARCINOMAS

Ovarian serous carcinoma is the most common and lethal type of ovarian cancer and its molecular etiology remains poorly understood. The expression of selected genes from the pRb pathway that regulates G_1_-S-phase progression, including cyclin D1, p16^INK4A^, cyclin E, p27^KIP1^, p21^WAF1/CIP1^, and p53, was examined in a consecutive series of 134 serous epithelial ovarian cancers (EOC) using immunohistochemistry. Molecular markers predictive of reduced overall survival in univariate analysis were overexpression of cyclin D1 (P = 0.03) and p53 (P = 0.03) and reduced expression of p27^KIP1^ (P = 0.05) and p21^WAF1/CIP1^ (P = 0.02), with the latter three also being prognostic for a shorter progression-free interval. In addition, patients displaying overexpression of p53 with concurrent loss of p21^WAF1/CIP1^ had a significantly shorter overall (P = 0.0008) and progression-free survival (P = 0.0001). On multivariate analysis, overexpression of cyclin D1 and combined loss of p21^WAF1/CIP1^ in the presence of p53 overexpression were independent predictors of overall survival. Similarly, the combination of p21^WAF1/CIP1^ loss and p53 overexpression was independently predictive of a shorter progression-free interval. Overexpression of p53 and cyclin E and reduced expression of p27^KIP1^ and p21^WAF1/CIP1^ were significantly associated with increasing tumor grade. This study confirms that dysregulation of cell cycle genes is common in EOC, and that aberrant expression of critical cell cycle regulatory proteins can predict patient outcome in serous EOC [1].

Buchynska *et al.* have shown that serous ovarian carcinomas are characterized by high proliferative activity (PI Ki-67 = 30.0 +/- 0.3%), p53 and p16^INK4A^ overexpression and low expression of p21^WAF1/CIP1^. The association between expression of these markers and ovarian tumor grade was defined: the maximal level of Ki-67, p53 and p16^INK4A^ and minimal of p21^WAF1/CIP1^ expression were observed in G3 tumors. So, low p21^WAF1/CIP1^ expression combined with p16^INK4A^ overexpression is considered to be a factor for a poor prognosis in serous ovarian cancer [2]. 

Reduced expression of the cyclin-dependent kinase inhibitor p27^KIP1^ has been reported to be associated with poor prognosis in several human cancers. In serous ovarian cancers, positive p27^KIP1^ staining rate was significantly higher in early stage than that in advanced stage diseases (p=0.030, Fisher’s exact test). Log-rank testing showed that negative p27^KIP1^ expression significantly correlates with poor survival in serous ovarian cancer patients (p=0.041). These results suggest that the underexpression of p27^KIP1^ caused by post-translational mechanism may contribute to the development and progression and result in poor prognosis of serous ovarian cancers [3].

It is well known that somatic mutation of p53 represents the most common molecular genetic alteration occurring in epithelial ovarian carcinoma. Inactivation of p53 was detected in 30-80% of ovarian carcinoma [4, 5].

Although p53 overexpression was a common feature of both mucinous and serous borderline tumors, p21^WAF1/CIP1^ overexpression appeared specific to serous tumors [6].

Statistical analyses showed a significantly higher expression of p53 in histologically high-grade tumors (grades 2 and 3), mainly of the serous subtyp. A statistical tendency toward higher expression of p53 in older patients (P= 0.08) was also observed. p53 is associated with serous carcinoma, loss of differentiation, and older patients. These results are in keeping with different pathogenetic pathways in subtypes of ovarian carcinoma, prompting the search for new strategies of prevention and treatment[7].

The frequency of p53 mutation in early-stage ovarian carcinomas of serous histology is comparable to that reported for advanced-stage tumors, and it is therefore likely to occur early in the progression of the most common his-tological variant of ovarian carcinoma [8]. For invasive carcinomas, the rate of mutation and expression increases with increasing tumor grade and stage, and is more common in tumors of serous histology [9]. Palazzo *et al.* demonstrated that coexpression of p21^WAF1/CIP1^ and MDM2 characterizes serous borderline tumors of the ovary and their implants, which suggests that these cell cycle control proteins are important in these tumors and may be related to tumor progression. Low expression of p53 protein in serous borderline tumors might be in part mediated by MDM2. This suggests that the p53 pathway is intact in most of these tumors, in contrast with carcinomas, in which high expression of p53 has been related to mutations of this gene [10].

## MUCINOUS CARCINOMAS

Mucinous carcinoma of the ovary accounts for 7% to 14% of all primary EOC [11]. Distinction of primary ovarian epithelial tumors from metastatic adenocarcinomas is challenging for tumors exhibiting mucinous, endometrioid, or mixed endometrioid/mucinous differentiation. Metastatic carcinomas with these types of differentiation can be derived from several sites, including the gastrointestinal tract and the uterus. Most endocervical adenocarcinomas exhibit muci-nous and/or endometrioid differentiation; they infrequently metastasize to the ovaries but may simulate primary ovarian tumors (both borderline and carcinoma). Most are high-risk human papillomavirus (HPV)-related and demonstrate diffuse p16 over-expression due to complex molecular mechanisms by which high-risk HPV transforming proteins interact with cell cycle regulatory proteins. Vang *et al.* evaluated this expression pattern for identifying metastatic endocervical adenocarcinomas in the ovaries among primary ovarian tumors and other metastatic adenocarcinomas having muci-nous and/or endometrioid/endometrioidlike differentiation. Immunohistochemical expression of p16 was assessed in 195 tumors, including 102 primary ovarian tumors (51 mucinous, 47 endometrioid, and 4 mixed mucinous-endometrioid tumors), 82 metastatic adenocarcinomas of known primary sites (colorectum: 34, endocervix: 19, pancreaticobiliary tract: 17, appendix: 7, stomach: 5), 11 metastatic adenocarci-nomas of unknown origin, and 4 adenocarcinomas of uncertain origin. Mean and median p16 expression values for HPV-positive endocervical adenocarcinomas were substantially higher than those for primary ovarian mucinous and endometrioid tumors, HPV-unrelated endocervical adenocar-cinomas, metastatic adenocarcinomas of unknown origin, and adenocarcinomas of uncertain (primary ovarian vs. me-tastatic) origin. Only the 15 HPV-positive endocervical ade-nocarcinomas and 6 other tumors had values of 80% or greater. Diffuse (>75% positive tumor cells), moderate to strong p16 expression is a sensitive (100%) and specific (97%) marker for identifying HPV-related endocervical ade-nocarcinomas metastatic to the ovary among the primary ovarian tumors and metastatic adenocarcinomas from other sites that are in the differential diagnosis of ovarian tumors having mucinous and/or endometrioid/endometrioidlike differentiation. p16 is useful as part of a panel of immunohisto-chemical markers for distinguishing primary ovarian tumors from metastases and, when diffusely positive, can suggest the cervix as a potential primary site for metastatic adenocar-cinomas of unknown origin [12].

Patients with advanced mucinous EOC have a poorer response to platinum-based first-line chemotherapy compared with patients with other histologic subtypes of EOC, and their survival is worse. Specific alternative therapeutic approaches should be sought for this group of patients, perhaps involving fluorouracil-based chemotherapy [13].

## ENDOMETRIOID CARCINOMAS

Plisiecka-Halasa *et al.*evaluated the clinical and biological significance of p21^WAF1/CIP1^, p27^KIP1^, c-myc, p53 and Ki67 expressions in ovarian cancer patients. Immunohisto-chemical analysis was performed on 204 ovarian carcinomas of International Federation of Gynecology and Obstetrics (FIGO) stage IIB to IV treated with platinum-based chemotherapy. Endometrioid and clear cell carcinomas differed from other carcinomas by having a low incidence of p53 accumulation, a high incidence of c-myc overexpression (70%) and a low median Ki67 labeling index (LI) (all with P <0.001). The authors have shown an independent predictive value of p21^WAF1/CIP1^ LI in ovarian carcinoma patients. The prognostic value of p21^WAF1/CIP1^ and p21^WAF1/CIP1^ plus p27^KIP1^ LI was determined by p53(-) status. A high frequency of c-myc overexpression in endometrioid and clear cell carcinomas may suggest its role in the development of these tumor types [14]. High-level p16 expression was observed in serous and endometrioid phenotypes, with a positive relation to high levels of both cell proliferation and p53 abnormalities. None of 131 cases analyzed by Saegusa *et al.* showed a methylation status of the p16 gene promoter [15]. Two different studies also revealed no evidence of methylation and low levels of mutations [16] [17]. However there are some data supporting p16 promoter hypermethylation as a mechanism underlying the downregulation of the gene. Milde-Langosch *et al.* found hypermethylation in 12/19 negative cases, most of them mucinous and endometrioid carcinomas [18]. Alterations in p15** were observed in serous, endo-metrioid and clear cell but not in mucinous carcinomas, suggesting that inactivation of p15 may be the histological type-specific event in ovarian tumorigenesis [19].

## CLEAR CELL CARCINOMAS

Ovarian clear cell carcinoma revealed significantly increased cyclin E associated with an increase in p21^WAF1/CIP1^, compared to the other histological subtypes [20].

Clear cell carcinoma revealed such trends as low expression of both p53 and cyclin A and significantly increased expression of both p21^WAF1/CIP1^ and cyclin E compared with the other histologic subtypes. In all ovarian carcinomas, a very strong positive correlation between p53 positive staining and cyclin A positive staining and a weak positive correlation between p21^WAF1/CIP1^ positive staining and cyclin E positive staining were recognized at the level of expression of cell cycle regulatory molecules. Clinical stage was the only independent predictive factor for the survival of the patients. Among ovarian adenocarcinomas, clear cell carcinoma exhibits a unique pattern of expression of cell cycle regulatory molecules, though in this study the survival did not correlate with histologic subtype, only with clinical stage [20].

Cyclin E expression is significantly higher in clear cell carcinoma than in serous carcinoma and is significantly related with p53 positivity [21].

Akahane *et al.* demonstrated that some genetic alterations, which induce p53 mutations in endometriosis, may affect malignant transformation of endometriosis into ovarian clear cell carcinoma [22].

## UNDIFFERENTIATED CARCINOMAS

The molecular changes in undifferentiated carcinomas of the ovary remain largely unknown [23].

In a recent study carried out by Rosen *et al.* overexpres-sion of cyclin E was found in 63.2% of the 405 primary ovarian carcinomas analyzed and was associated with clear cell, undifferentiated, and serous carcinoma (P < or = .001), high-grade tumors (P < or = .001), late-stage disease (P = .002), age older than 60 years at the time of diagnosis (P = .04), and suboptimal cytoreduction (P = .001). A high percentage of cyclin E-expressing cells was associated with a poor outcome in univariate and in multivariate analyses. In addition, cyclin E levels also reduced survival in the late-stage disease group and in patients who underwent suboptimal debulking. Cyclin E was identified as an independent prognostic factor in patients with ovarian carcinoma. The accumulation of cyclin E protein may be a late event in tu-morigenesis and may contribute to disease progression in these patients [24]

## CONCLUSIONS

The identified molecular changes in epithelial ovarian cancers can facilitate the rational development of new diagnostic modalities and tailored therapies for these malignan-cies. Most of the G_1_-S regulators play an important role in ovarian cancer due to the fact that they control the G_1_-S transition, a crucial step in cell cycle control. Cyclin E is a key regulator of the G_1_-S transition. Abnormalities in cyclin E expression have been related to survival in a variety of cancers. Cyclin E plays an important role in ovarian carcino-genesis of clear cell, undifferentiated, and serous carcinomas and its overexpression may be an indicator of a poor prognosis. Cyclin E expression is significantly higher in clear cell carcinoma than in serous carcinoma and is significantly related with p53 positivity. Overexpression of cyclin E was also found to be associated with undifferentiated carcinomas of the ovary. The actual mutation of p53 is the most common molecular alteration occurring in both early-stage and invasive ovarian carcinomas, especially in those of serous histology, and confers resistance to chemotherapy and lead to shortened overall survival. Epithelial ovarian tumors showing p53 alterations are significantly less sensitive to chemotherapy and more aggressive than those with functional p53 and overall survival is shortened in patients with p53 mutations [25, 26]. In serous carcinomas the combination of p21^WAF1/CIP1^ loss and p53 overexpression was independently predictive of a shorter progression-free interval. Coexpres-sion of p21^WAF1/CIP1^ and MDM2 characterizes serous borderline tumors of the ovary and their implants, which suggests that these cell cycle control proteins are important in these tumors and may be related to tumor progression. Low p21^WAF1/CIP1^ expression combined with p16 overexpression is considered to be the indicator for a poor prognosis in serous ovarian cancer.

Mucinous and endometrioid carcinomas are largely characterized by p16 downregulation and the gene promoter hy-permethylation. Lack of p16 is associated with p53 wt (wild type) and is typical of mucinous and endometrioid tumors. Little is known about the role of p15 in ovarian carcinomas. No mutations have been detected. Its inactivation may be the histological type-specific event in ovarian tumorigenesis. Alterations in p15 gene occur in serous, endometrioid and clear cell carcinomas but not in mucinous carcinomas. There are also a number of studies reporting no correlation between the expression of cell cycle genes and the different histo-types of ovarian carcinoma.

In fact, cyclin D1 and c-Myc which are key participants in the cell-cycle pathway are frequently overexpressed in epithelial ovarian carcinomas, but they are not correlated with a particular histologic subtype [27] and p27^KIP1^ subcel-lular location in the cytoplasm was independently associated with poorer survival among women with ovarian carcinoma, particularly for those with late-stage disease and regardless of tumor histotype[28].

**Fig. (1):**
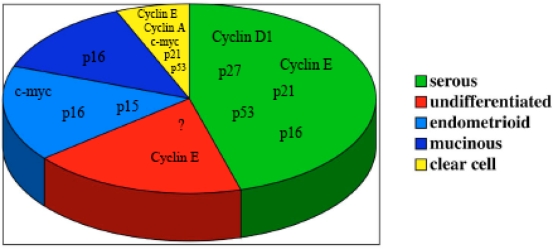
Schematic model of cell cycle regulator genes involved in carcinogenesis of the different histotypes in epithelial ovarian cancer. The question mark in the undifferentiated tumor section underlines that the role of cell cycle genes in this histotype of ovarian cancer is largely unknown.

The described abnormalities of cell cycle regulators in ovarian carcinomas (Fig. 1) suggest that most of the cell cycle regulatory genes play a crucial role in ovarian carcinoma tumorigenesis and/or development. The main goal in cancer therapy remains an early diagnosis of the disease, and some of the cell cycle genes described could be useful markers for achieving this goal, and therefore more targeted therapies.

## GLOSSARY

BRCA1 and BRCA2: Tumor suppressor genes
Cyclins: Family of genes involved in the progression of cells through the cell cycle
Cyclin E: Gene that regulates cell cycle. Cyclin E is one of the key regulators of the G_1_/S transition in the cell cycle. Cyclin E binds to G_1_ phase Cdk2, which is required for the transition from G_1_ to S phase.
Cyclin D Family: Family of three closely related genes termed cyclin D1, D2 and D3 that are expressed in an overlapping redundant fashion in all proliferating cell types and collectively control the progression of cells through the cell cycle
Cyclin D1: Gene that belongs to Cyclin D family of genes which function as the regulatory subunits of cyclin/cyclin dependent kinase (Cdk) holoenzymes that regulate entry into and progression through the cell cycle
CDK 2 (Cyclin-Dependent Kinase 2): Gene that regulates cell cycle. CDK2 is a member of a large family of protein kinases that initiates the principal transitions of the eukaryotic cell cycle
CDK4 (Cyclin-Dependent Kinase 4): Gene that regulates cell cycle. The complexes formed by CDK4 and the D-type cyclins are involved in the control of cell proliferation during the G1 phase. CDK4 is inhibited by p16^INK4a^.
CDK 6 (Cyclin-Dependent Kinase 6): Gene that regulates cell cycle. The activity of this kinase first appears in mid-G_1_ phase, which is controlled by the regulatory subunits including D-type cyclins and members of INK4 family of CDK inhibitors. This kinase, as well as CDK4, has been shown to phosphorylate, and thus regulate the activity of tumor suppressor protein pRb
Cyclin A: Gene that regulates cell cycle. Cyclin A binds to S phase Cdk2 and is required for the cell to progress through the S phase of cell cycle
Cyclin B: Gene that regulates cell cycle. Cyclin B is a mitotic cy-clin. The amount of cyclin B (which binds to Cdk1) and the activity of the cyclin B-Cdk complex rise through the cell cycle until mitosis, where they fall abruptly due to degradation
c-myc: Oncogene overexpressed in a wide range of human cancers
INK4 Family: Family of cyclin-dependent kinase inhibitors
KI-67: Gene associated with cell proliferation
MDM2: Target gene of the transcription factor tumor gene p53; its overexpression inhibits p53 gene.
p53: Tumor suppressor gene
p27^KIP1^: Tumor suppressor gene. p27^KIP1^ belongs to the family of cell cycle regulators called cyclin-dependent kinase inhibitors (CDKI), which bind to “cyclin-CDK” complexes and cause cell cycle arrest in the G^1^ phase.
p21^WAF1/CIP1^: Tumor suppressor gene. This gene encodes a potent cy-clin-dependent kinase inhibitor. The encoded protein binds to and inhibits the activity of cyclin-CDK2 or -CDK4 complexes, and thus functions as a regulator of cell cycle pro-gression at G_1_. The expression of this gene is tightly controlled by the tumor suppressor protein p53, through which this protein mediates the p53-dependent cell cycle G_1_ phase arrest in response to a variety of stress stimuli
Rb (Retinoblastoma Gene): Tumor suppressor gene. Rb prevents the cell from replicating damaged DNA by preventing its progression through the cell cycle into its S (synthesis phase) or progressing through G_1_ (first gap phase)
pRb (Retinoblastoma Protein) Pathway: The pRb pathway plays a key role in controlling the G_1_/S transition in cell cycle progression
p16^INK4a^: Gene that belongs to INK4 gene family. p16 is a tumor suppressor gene
p15^INK4b^: Gene that belongs to INK4 gene family. p15INK4b is a specific inhibitor of cdk4/cdk6 and is periodically expressed during cell cycle
